# Influence of occupational safety culture on the occupational risk level in the organization

**DOI:** 10.3389/fpubh.2025.1595869

**Published:** 2025-06-25

**Authors:** Pavlo Saik, Vitalii Tsopa, Larysa Koriashkina, Serhii Cheberiachko, Oleg Deryugin, Vasyl Lozynskyi

**Affiliations:** ^1^Belt and Road Initiative Center for Chinese-European Studies (BRICCES), Guangdong University of Petrochemical Technology, Maoming, China; ^2^MIM-Kyiv, Kyiv, Ukraine; ^3^Dnipro University of Technology, Dnipro, Ukraine

**Keywords:** safety, risk, safety culture, occupational injuries, health, hazardous event, incident, attitude to safety

## Abstract

**Introduction:**

This study aims to develop a methodology for assessing the risk of hazardous situations, incidents, and related events, with consideration of the level of “occupational safety culture” among employees within an organization.

**Methods:**

The research employs a systematic approach, incorporating mathematical and simulation modeling to evaluate the influence of safety culture on occupational risk levels.

**Results:**

A methodology has been developed to assess the safety culture level and its impact on the likelihood of hazardous events. This methodology is considering based on the degree of compliance with Occupational Safety and Health Management Systems (OHSMS) requirements by employees, categorized into five stages of safety culture: (1) indifference, (2) response, (3) dependence, (4) independence, and (5) interdependence. Key factors influencing each stage have been identified. The model demonstrates that employees’ compliance with OHSMS requirements is shaped by the roles of managers, safety professionals, and employees within a systemic and social interaction framework.

**Discussion:**

The proposed model enhances the occupational risk management process by incorporating safety culture as a key factor. It establishes a correlation between the level of safety culture and the risk of incidents at different development stages. The model also highlights how initial compliance levels, awareness of safety requirements, and peer and managerial influence affect risk outcomes. Furthermore, it identifies three primary causes underlying non-compliance: negligence, lack of competence (due to insufficient training), and selfish motives.

## 1 Introduction

Occupational safety should be a top priority for modern organization. Analysis of high-profile events, such as the ammonium nitrate blast in Beirut (1), the accident at the Fukushima Daiichi nuclear power plant (2, 3), the Deepwater Horizon oil spill (4), the Space Shuttle Challenger disaster (5) and the Chernobyl disaster (6), indicates the constant presence of threats in our lives, which, despite all the protection systems implemented in complex systems, can, under certain conditions, lead to the events mentioned (7). All emergencies and accidents, without exception, are a source of information, lessons to be learned in order to prevent their occurrence in the future. In particular, they have led to a certain rule that disasters and accidents are the result of many small failures or errors, faulty systems, and inappropriate organizational conditions (8, 9). This requires a constant search for a balance between the organization’s activity (productivity, desire for maximum profit, etc.) and the creation of conditions for employees to comply with occupational safety and health protection requirements in the workplace in order to avoid accidents, emergency situations, etc. due to the accumulated failures and errors (10–14).

To address this challenge, enterprises implement risk management systems, which form the foundation of occupational health and safety (OHS) management (8). The accuracy and reliability of risk assessment are critical for the effectiveness and efficiency of decisions aimed at reducing the likelihood of hazardous events.

Qualitative and semi-quantitative risk assessment models are the most commonly used in organizations (9). Most of these models rely on expert judgment, which can vary significantly and thus affect the precision of the results (10). To overcome this limitation, several approaches have been proposed. One effective method involves multifactor analysis of hazardous factors that increase the likelihood of adverse events (11, 12). This approach requires the investigation of different categories of risk factors, including organizational, technological, technical, social, and economic. Among these, a particularly important category includes factors that define the level of safety culture within the organization. This culture reflects employees’ attitudes toward complying with OHS requirements. It is well established that a responsible attitude toward OHS significantly reduces the number of errors that may lead to injuries or occupational diseases (13). As such, an essential element of risk management is identifying the current stage of the organization’s safety culture development and understanding its impact on occupational risk.

This raises a critical research task: establishing the relationship between the development of safety culture—determined by employee attitudes toward fulfilling OHS responsibilities—and its influence on occupational risks. The goal is to provide a scientific rationale for preventive and protective strategies aimed at reducing failures, errors, system malfunctions, and other incidents that may lead to dangerous situations.

A review of existing literature highlights a persistent challenge in enhancing the reliability of risk assessments in organizations (15–18). Summarizing the issues discussed in these studies, it becomes evident that cognitive gaps in employee surveys and the need to integrate organizational and technical aspects of production processes into risk assessment are common concerns. Some researchers (16, 18, 19) emphasize the potential of “reverse engineering” the structure of risk assessment tools to enhance reliability. Others advocate for the use of quantitative risk assessment methods (17, 20, 21). Furthermore, several experts argue for incorporating the influence of organizational culture through expert-defined weighting coefficients (19, 22–24).

It is interesting that most recent publications have focused on developing the safety culture maturity models in organizations (25, 26). It is believed that this will help shape the vector of their development and utilize new opportunities. Thus, the authors of the paper (27) have analyzed 41 scientific papers describing safety culture maturity models, of which 12 provide an appropriate level of confidence in the results obtained. This makes it possible to identify key indicators for assessing the safety culture level. It is interesting that most researchers in the study of safety culture maturity pay attention to the level of employee training organization, management commitment, as well as employee commitment and involvement in the OH&S risk management process (28–30). For some reason, however, the revised works do not indicate the need to assess the flexibility of the OH&S risk management process (31) to take into account the time frame of changes in the same safety culture or organizational culture.

In summary, the review of scientific literature suggests that safety culture represents a combination of organizational, social-psychological, and environmental values (32). It comprises intangible requirements regarding workplace safety and its components (33), collective perceptions and attitudes (safety climate) (34), and shared behavioral norms and artifacts (35) that reflect the organization’s worldview through its mission and vision. Thus, safety culture encompasses a range of hazardous factors—organizational, psychosocial, and communicational—that directly or indirectly influence the level of occupational risk (36–40). Consequently, there is a need to develop an effective mechanism to account for its impact on risk magnitude.

Accordingly, the aim of this article is to enhance the effectiveness of the enterprise risk management system by developing a methodology for occupational risk assessment that incorporates the level of safety culture, as determined by employees’ attitudes toward complying with occupational health and safety (OHS) requirements.

## 2 Materials and methods

**Figure 1:**
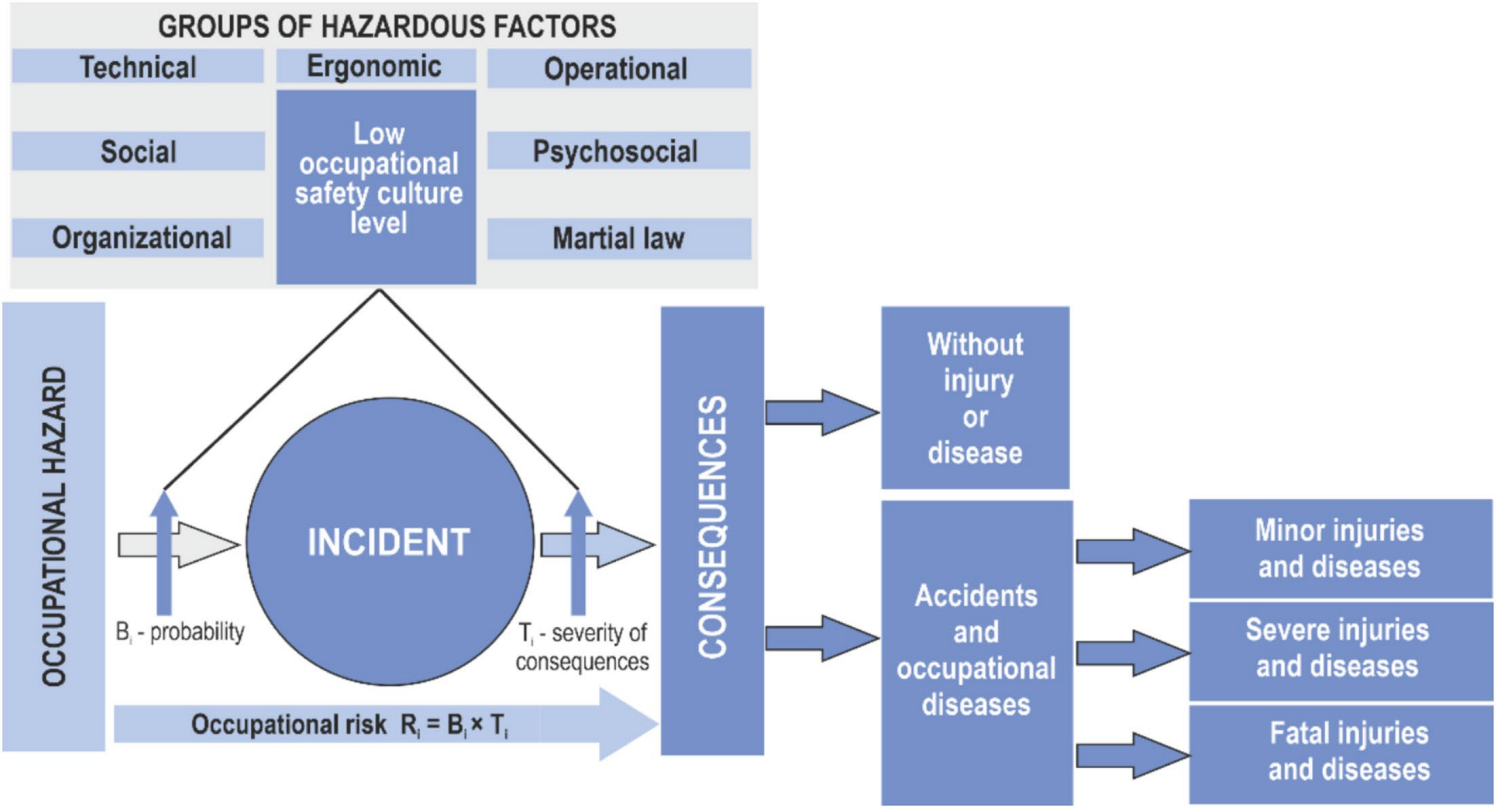
Occupational risk management model taking into account a group of hazardous factors of the “occupational safety culture” level in an organization.

To support planning of the occupational risk management process, an enhanced bow-tie model is proposed (Figure 1), based on IEC 31010:2019. This model illustrates the relationship between occupational hazards (workplace threats), hazardous events (injuries or occupational diseases), and their health consequences for workers (mild, moderate, or severe) (27, 41). The likelihood of a hazardous event is influenced by various groups of hazardous factors, including organizational, technical, operational, social, ergonomic, psychosocial, and those related to military conditions. In addition to these, safety culture factors—reflecting the attitudes of managers, safety professionals, and workers toward compliance with occupational health and safety (OHS) requirements—must be considered. Accident causation analysis (28, 29, 42) demonstrates that noncompliance with OHS requirements by employees is often a direct result of an underdeveloped safety culture (34). Identifying hazardous factors characteristic of a specific safety culture allows organizational leadership to assess the potential for achieving the goal of reducing occupational risks to an acceptable level—under the condition that all employees fully adhere to OHS requirements.

Furthermore, the indicator of compliance with OHS requirements provides a more precise estimation of occupational risk, assuming that all control measures are fully implemented due to a high level of safety culture within the organization (43).

A five-step algorithm is proposed for risk assessment. The first step *“Identification of occupational hazard risk components”* involves studying the working conditions in the workplace to identify the presence of various occupational hazards. This will make it possible to identify cause-and-effect relationships between an occupational hazard—a hazardous event (incident, accident, emergency situations, occupational disease, etc.) and consequences (loss of life or health of workers). Different methods of information gathering are used to conduct this step. For example, documentation analysis, observation, experimentation, accident statistics, etc. (41, 44).

The second step *“Identification of hazardous factors”* aims to identify all hazardous factors of the internal environment that influence the probability of a hazardous event occurring from previously identified hazards. This can be done using methods such as SWOT analysis, PEST analysis or PIMS analysis, questionnaires, observation, employee surveys, etc. (42–45).

Scientific and technical literature identifies the following groups of internal hazardous factors that increase the injury rate and the number of occupational diseases: organizational, technical, operational, social, ergonomic, psychosocial and military (41, 46). Analysis of the causes of injuries (47–51) indicates the need to add one more group, which is related to the level of non-compliance with occupational safety requirements by employees of the organization and characterizes the low level of safety culture development (52). Identification of hazardous factors characteristic of the safety culture will allow the organization’s management to understand the possibilities of achieving the goal of reducing occupational risks to an acceptable level while fully complying with OH&S requirements of employees. In addition, the level of compliance with OH&S requirements provides an opportunity to specify the occupational risk level, which is calculated on the assumption that all control measures are fully implemented (53).

A register is compiled for each group of hazardous factors. The safety culture group can include a significant number of different factors characterizing the attitude of management and employees to occupational safety, the values formed in the organization, occupational safety training, leadership, climate, development of occupational safety policy, implementation of occupational risk assessment procedure, and others (54, 55).

At the third step *“Analysis of occupational hazard risks from hazardous factors,”* the level of occupational hazard risk is calculated as the sum of occupational risks from all identified hazardous factors related to the given hazard (37).

At the fourth step *“Assessing the occupational risk level*,” the level of occupational risks is assessed as acceptable, acceptable with verification or unacceptable. Any qualitative or quantitative scale can be used, according to the objectives set by the organization’s management and financial capabilities.

At the last step *“Risk processing,”* the assessment of occupational risk levels is documented. In the event of an unacceptable occupational risk level, return to the stage of analyzing occupational hazards and hazardous factors with the development of precautionary and protective measures to reduce it. Then follow the above steps again.

The analysis of literary sources (42, 56) has identified a relationship between the safety culture level and the number of incidents through the level of compliance with OH&S requirements of employees. In this case, the level of compliance with OH&S requirements of employees is one of the important characteristics of safety culture (as a general concept of commitment and personal responsibility of all persons in an organization engaged in any activity affecting the occupational risk level) (57, 58), which depends on employees’ awareness of the need to comply with occupational safety requirements (48, 49). At the same time, the awareness of employees to comply with the rules is influenced by the authority of organization’s manager and his attitude towards safety (50, 58), the influence of informal leaders in a particular unit (59), the influence and attitude of those responsible (specialists) for occupational safety regarding the conscientious performance of their duties (60). Also, awareness is influenced by providing occupational safety training, introducing feedback, monitoring systems, reporting, etc.

**Figure 2:**
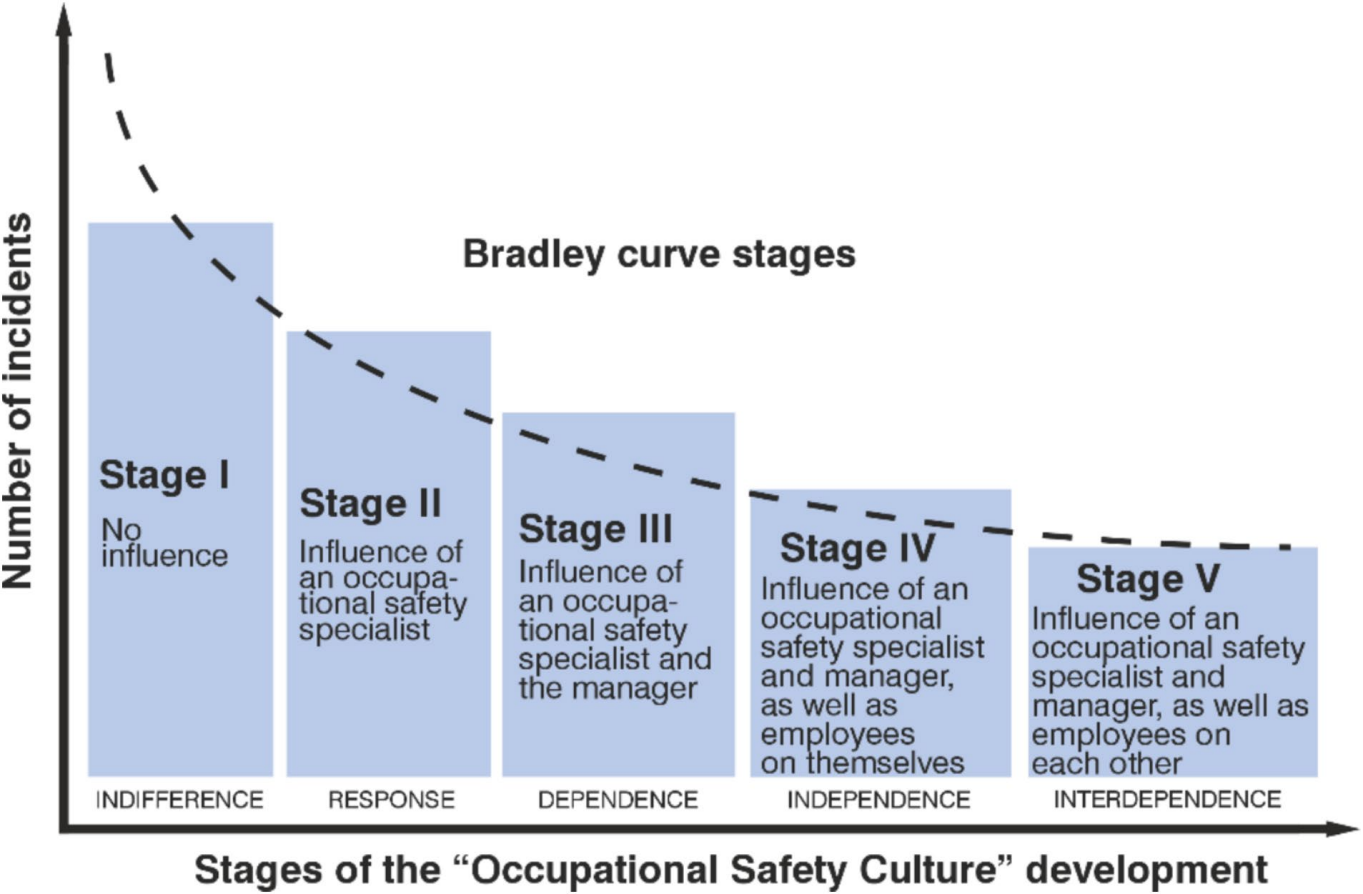
Stages of the model for “occupational safety culture” development.

The level of safety culture can be effectively represented using the Bradley Curve (Figure 2), which provides a descriptive characterization of the current organizational state (2), particularly when behavior-based safety (BBS) programs are implemented (61). This curve helps illustrate the relationship between safety culture and compliance with occupational health and safety (OHS) requirements (38, 44), as it reflects employees’ attitudes toward safety. The Bradley Curve is considered particularly suitable for guiding organizations through transitions from lower to interdependent stages of safety culture maturity (62). However, when describing highly developed safety cultures, the Bradley Curve alone may be insufficient due to inconsistencies in its evaluative parameters (63). To address these limitations, DuPont introduced the DuPont Integrated Safety Approach, which builds upon the concept of visible leadership to initiate and sustain meaningful and lasting cultural change at all stages of development (38). Despite its shortcomings, the Bradley Curve remains a useful tool for illustrating the correlation between employee attitudes toward OHS compliance and the number of incidents—allowing for preliminary estimation of the likelihood of hazardous events.

To identify the key factors characterizing various levels of safety culture maturity, both mathematical and simulation modeling techniques are widely applied.

According to Bradley curve, consider five levels of safety culture development: indifference, response, dependence, independence, interdependence, which can be characterized by four key indicators (the influence of managers, specialists and employees themselves) on each other regarding the compliance with OH&S requirements of employees, as well as the influence of their self-awareness, reflecting their relationship with the stages of safety culture development (Table 1).

**Table 1:** Relationship between safety culture factors and its development levels.

No	Safety culture factor	Name of the stages of safety culture development				
		Indifference	Response	Dependence	Independence	Interdependence
	Influence on the compliance with the OHSMS requirements by employees					
		I	II	III	IV	V
1	Influence of safety specialists on the compliance with the OHSMS requirements by employees	Indifference	Non-indifference	Non-indifference	Non-indifference	Non-indifference
2	Influence of managers on the compliance with the OHSMS requirements by employees	Indifference	Indifference	Non-indifference	Non-indifference	Non-indifference
3	Influence of employees on themselves (self-awareness) regarding compliance with the OHSMS requirements	Indifference (on the basis of instincts)	Indifference	Indifference (on the basis of instincts)	Non-indifference	Non-indifference
4	Influence of employees on each other regarding compliance with the OHSMS requirements	Indifference	Indifference	Indifference	Indifference	Non-indifference

Red - Indifference, Green - Non-indifference.

Moreover, each influence reflects a set of requirements fulfilled in different areas of safety culture: leadership, training, interaction, support, awareness, attitude, etc. (51). The level of influence will be characterized through employees’ attitudes towards compliance with occupational safety requirements (64), which are characteristic of each safety culture level.

**Figure 3:**
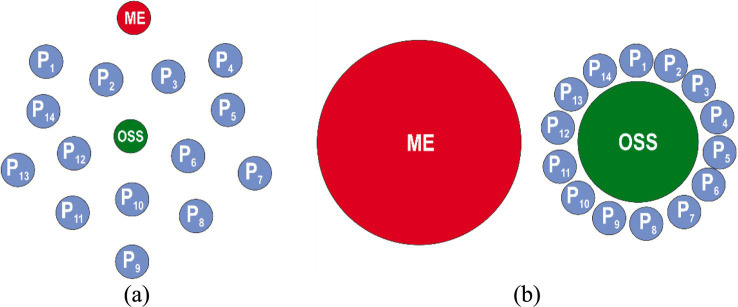
Stage I safety culture model: “indifference” represented by Euler model **(а)** and the Venn model **(b)**: ME—manager of the enterprise; OSS—occupational safety specialist; Р_1_-Р_14_—employees (blue color indicates a lack of interest in complying with safety requirements).

The first stage of occupational safety culture model is “indifference” to safety issues, where every employee instinctively seeks a safe approach to performing a production task. Managers and occupational safety specialists have a lot of issues related to the organization formation, while paying insufficient attention to the compliance of employees with occupational safety requirements (Figure 3).

This usually occurs at the stage of formation of an organization, when the goals are not yet clear, there is no process approach in management, and there is no focus on occupational safety issues. This stage involves the search for like-minded people, preparation for the implementation of the idea, legal registration of the organization, and recruitment of operational staff. In this case, the state of occupational safety is controlled on the basis of instincts, as there is a significant workload of managers who do not pay attention to the requirements of regulatory legal acts on labor protection issues, including due to limited financial resources. Employees, at the level of acquired reflexes, observe safety rules and, to a greater or lesser extent, instinctively do not violate them out of a sense of self-preservation rather than because they know them.

**Figure 4:**
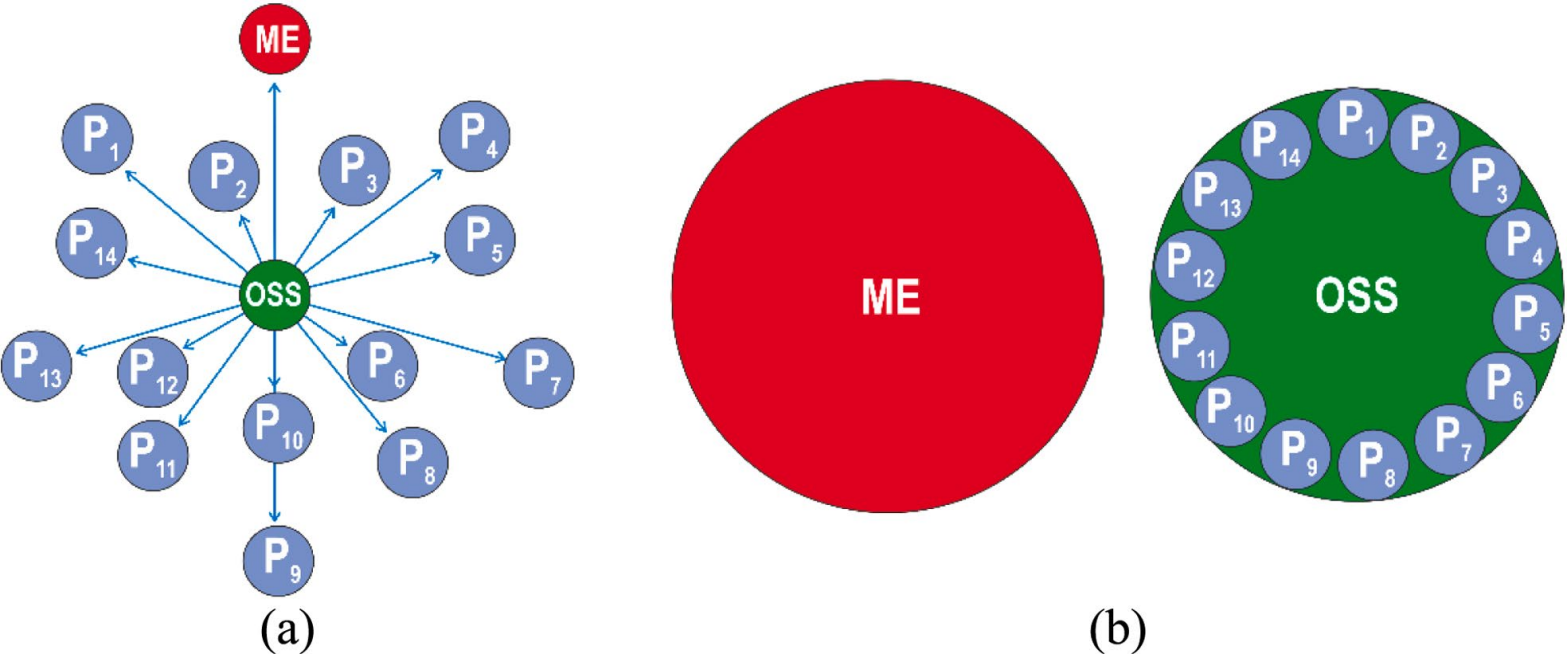
Stage II safety culture model: “response” represented by Euler model **(а)** and the Venn model **(b)**: ME—manager of the enterprise; OSS—occupational safety specialist; Р1-Р14—employees.

The second stage of occupational safety culture model is “response,” when each employee starts to follow certain safe approaches to performing a production task when forced to do so by occupational safety specialists (Figure 4).

It is characterized by a period of rapid enterprise growth, awareness of its mission and formation of a development strategy (informal communications and structure, high commitments and responsibility). In this case, management is afraid of inspections by supervisory authorities, reacts painfully to production stoppages and penalties.

**Figure 5:**
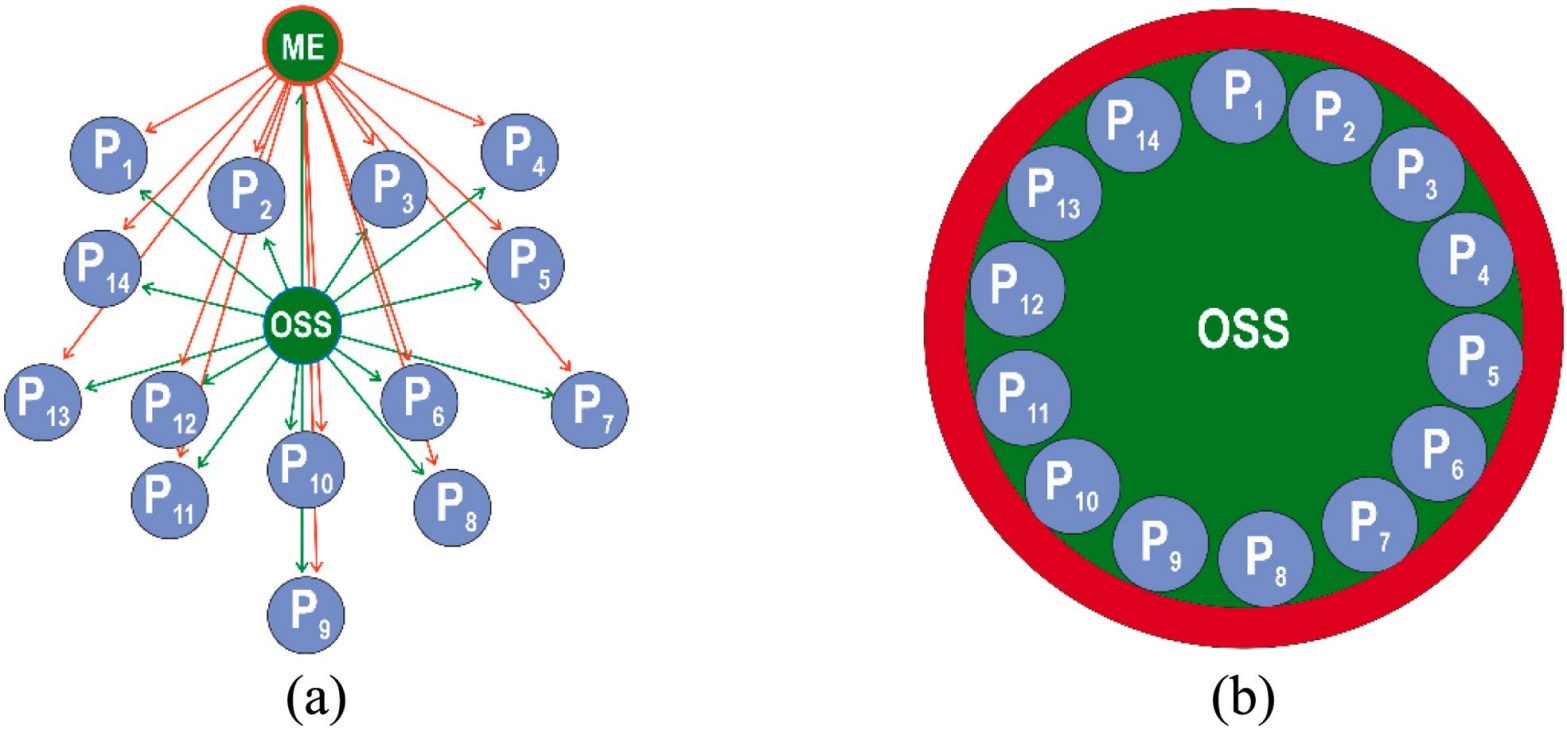
Stage III safety culture model: “dependence” represented by Euler model **(а)** and the Venn model **(b)**; ME—manager of the enterprise; OSS—occupational safety specialist; Р1-Р14—employees.

The third stage of occupational safety culture model is “dependence,” when each employee starts to follow certain safe approaches to performing a production task when forced to do so by both occupational safety specialists and the organization’s management (Figure 5).

This is a period of stabilizing growth to achieve a leading position for the organization in the market. In this case, management understands that there is occupational safety and health protection legislation that needs to be complied with. The same is required of subordinates. A systematic approach to employee occupational safety and health protection is formed, and best practices are introduced in the organization. Compliance with the requirements of regulatory legal acts on the issues of occupational safety and health protection of employees in the workplace is monitored. Funds and resources are allocated on a planned basis for occupational safety and health protection of employees, taking into account occupational risk levels. However, even though employees are formally trained, but if managers do not supervise them, they may violate safety rules in the absence of immediate hazard.

**Figure 6:**
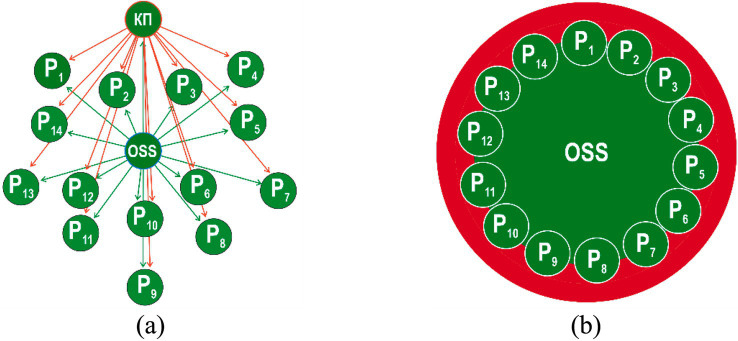
Stage IV safety culture model: “independence” represented by Euler model **(а)** and the Venn model **(b)**; ME—manager of the enterprise; OSS—occupational safety specialist; Р1-Р14—employees.

The fourth stage of occupational safety culture model is “independence” of the production task through employees’ awareness of the need to comply with occupational safety requirements and apply safe working methods (Figure 6).

At this stage, the organization continues to grow, and its activities are expanding. In this case, the management demonstrates by personal example its commitment to safety ideas, masters management tools (OH&S risk management, training, etc.), voluntarily implements international standards for occupational safety and health protection of employees, not stipulated by the legislation. Employees follow safety rules themselves, observe safe behavior in the workplace, but are indifferent to fulfilling the requirements of other employees. A system for managing the occupational health and safety of company employees is created and implemented based on international standards (for example, ISO 45001:2018), but only formally. Moreover, at the fourth stage, the occupational safety culture level is actively developed, where management sees occupational safety among the enterprise’s values and makes the same demands when choosing partners and contractors, pursues a policy of openness—is ready to share experience and best practices in the field of occupational safety and health protection of employees.

**Figure 7:**
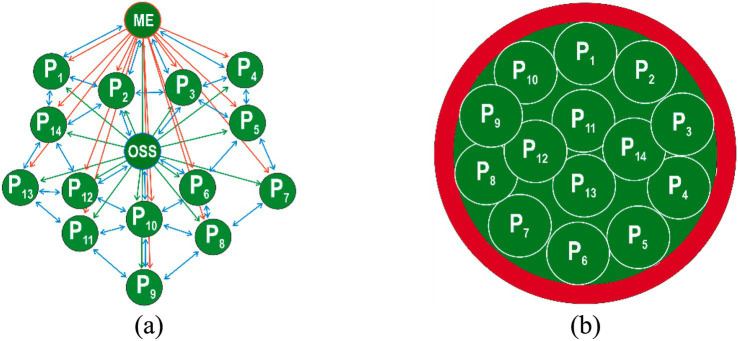
Stage V safety culture model: “interdependence” represented by Euler model **(а)** and the Venn model **(b)**; ME—manager of the enterprise; OSS—occupational safety specialist; Р1-Р14—employees.

The fifth stage of occupational safety culture model is “interdependence,” when each employee starts to follow certain safe approaches to performing a production task through awareness of the need to implement safe practices with the help and support of colleagues (Figure 7). The staff are genuinely proud to be part of a common cause—occupational safety, strive to work without injuries and work as one team, supporting and motivating each other to comply with the requirements.

**Figure 8:**
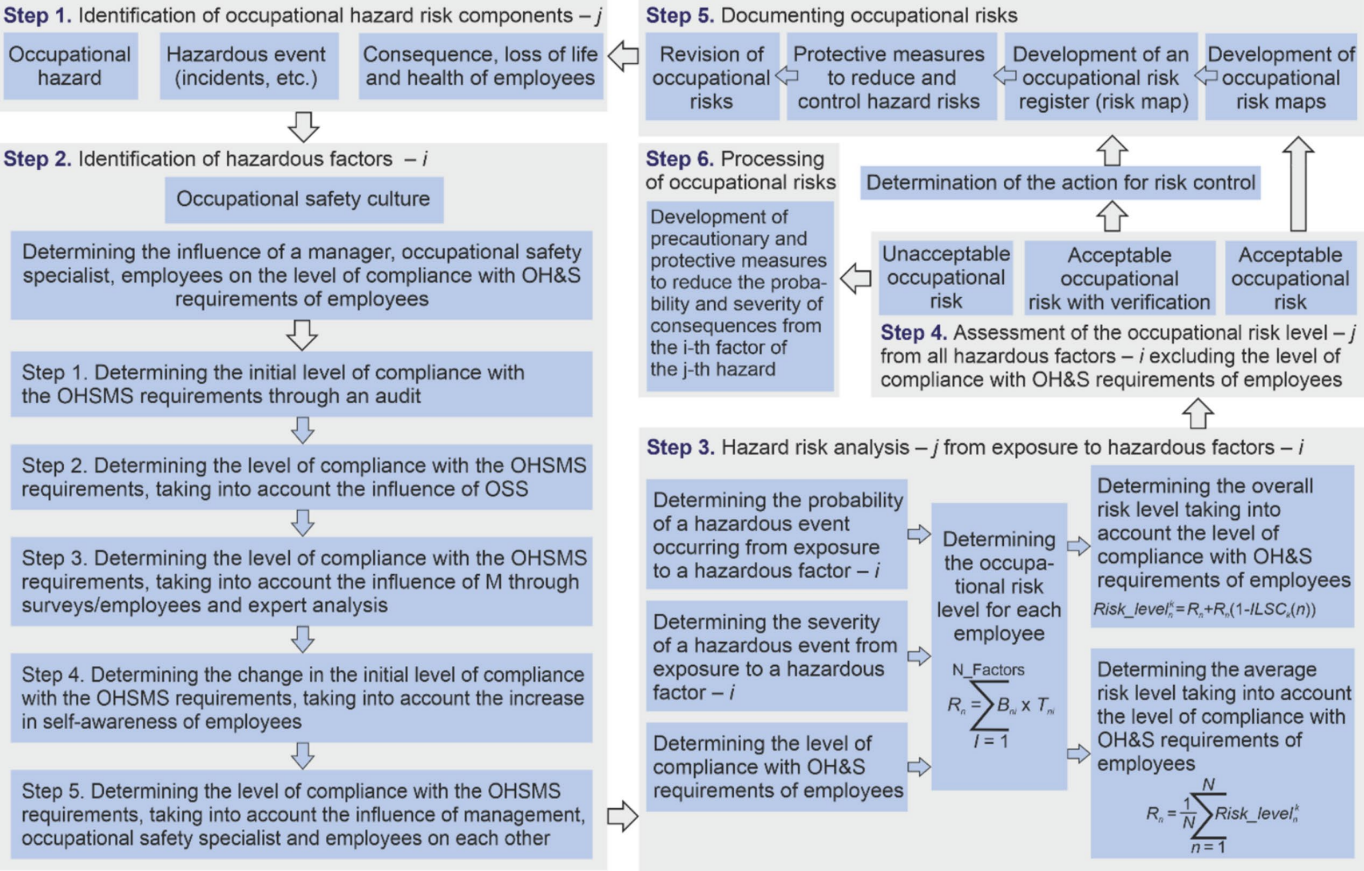
Occupational risk management process, taking into account the level of compliance with OH&S requirements of employees.

Thus, when determining the occupational risk level, the attitude of all employees and management to occupational safety issues will be taken into account through the level of compliance with OH&S requirements of employees. This leads to the need to add two additional steps to the above-described algorithm for determining the occupational risk level (Figure 8):

- To identify hazardous factors that are specific to the safety culture and are formed by the level of compliance with OH&S requirements of employees (step 2);
- Processing occupational risks through the implementation of precautionary and preventive measures to control the risk, in case of non-compliance with a certain risk level (step 6).

At the second step, to determine the level of compliance with OH&S requirements of employees, it is proposed to perform five stages to assess the fulfillment of employees’ requirements, taking into account the influence of the manager, occupational safety specialist and self-awareness.

*At the first stage,* the initial level of compliance with OH&S requirements of employees is determined particularly for each n -th employee, $n=\bar{1,N}$ , $q_{n}$ by conducting an appropriate occupational safety audit using generally acceptedpproaches (38, 63). For this purpose, it is recommended to use safety audit reports, questionnaires of which are developed based on the basic national requirements for occupational health and safety management system. It is recommended that the requirements of the ISO 45001 standard, which provides (ISO 19011:2018) seven groups (organizational context, leadership, planning, support, operation, performance evaluation, improvement) (an example is provided in Appendix 1) (51, 64). A fragment of the questionnaire for employees is presented in the Appendix. It is also possible to use already developed questionnaires that characterize in detail the attitude of each employee towards his/her duties, for example, the 18-point scale from Fernández-Muñiz (65), which has four subcategories, namely safety policy, safety training, communication with issues of precaution and preventive planning. Additionally, to determine the weighting coefficients for the influence of the occupational safety specialist, the unit manager, self-awareness, and mutual support among employees in complying with occupational safety requirements, questionnaires based on the BFI-10 format (65) can be used. These questionnaires assess the development level of five personality traits associated with conscientiousness in fulfilling duties, particularly those related to occupational safety compliance.

In this case, the $q_{n}$ level is calculated as the ratio of actually fulfilled requirements, determined in the course of the audit to the whole total set of requirements provided for in the occupational health and safety management system. Therefore, the level of compliance with OH&S requirements of the n -th employee at the first stage is determined by its initial value:


$$\mathrm{ILS}C_{1}(n)=q_{n},n=\bar{1,N}.$$

*At the second stage,* the level of compliance with OH&S requirements of employees is specified taking into account the influence of an occupational safety specialist through employee interviews and observations of their actions without the presence of an occupational safety specialist. This can be done using the questionnaires to determine the Global Adaptation Index (GAIN index) or using five-item Responsible Leadership Scale from Voegtlin (66) by summing the scores on a five-score Likert scale; the response scale ranges from 1 (completely disagree) to 5 (completely agree) (42, 55). Then the determined number of scores is converted into a relative index (from 0 to 1), using the set value to the maximum possible.

If to assume that the influence of the occupational safety specialist is determined not only by the degree of communication between the employee and the instructor, but also by the percentage $0\leq p_{1}<100$ with which, on average, employees listen to the advice or requirements of the instructor and change their attitude towards compliance with these requirements, then for each employee the individual level of compliance with OHSMS requirements at this stage of the enterprise development can be calculated by the following equation:


$$\mathrm{ILS}C_{2}(n)=\mathrm{ILS}C_{1}(n)+p_{1}q_{2}a_{2n}(1-\mathrm{ILS}C_{1}(n)),n=\bar{1,N}.$$

where $q_{2}$ is the expert initial assessment of the individual level of compliance with the OHSMS requirements by a safety specialist, $a_{2n}$ $n,n=\bar{1,N}$ is a numerical characteristic of the degree of his/her influence on the n -th employee, taking a value from 0 to 1.

It is noteworthy that methods from sociometry, psychology, and mathematical statistics can be applied to assess the strength of interpersonal connections and the influence of one employee on another within an organization. Sociometric studies make it possible to reveal the structure of interpersonal relationships within a team, including the perception of authority. Based on various observations, interviews, surveys, and psychological testing, interaction matrices (i.e., contact intensity matrices) are constructed, recording the frequency and types of interactions (e.g., work-related, informal, conflictual) between employees. The collected data are aggregated and analyzed using statistical techniques. For instance, the degree of trust and loyalty toward management or occupational safety specialists can be evaluated using Spearman’s rank correlation coefficient.

*At the third stage*, the level of compliance with the OHSMS requirements by employees is recalculated taking into account the influence of managers (administrative resources) (54) using the equation:


$$\mathrm{ILS}C_{3}(n)=\mathrm{ILS}C_{1}(n)+\frac{p_{1}s_{n}(1-\mathrm{ILS}C_{1}(n))}{100},$$

where $s_{n}=(q_{1}a_{1n}+q_{2}a_{2n}-q_{2}a_{2n}q_{1}a_{1n}),n=\bar{1,N}$ ; $s_{n}$ is level, reflecting the joint influence of the manager and the occupational safety specialist; $q_{1}$ is the expert initial assessment of the level of compliance with the OHSMS requirements and job responsibilities by a manager, $a_{1n}$ $n,n=\bar{1,N}$ , is a numerical characteristic of the degree of his/her influence on the n -th employee, taking a value from 0 to 1. Note that the value $s_{n}$ is calculated by analogy with the probability of the sum of joint events, taking into account that the manager and the occupational safety specialist may have the same administrative tools (reward/punishment) to influence the employee.

*At the fourth stage,* the presence of a conscious attitude of employees is considered, that is, $p_{1}=100\%$ ; and therefore,


$$\mathrm{ILS}C_{4}(n)=\mathrm{ILS}C_{1}(n)+s_{n}(1-\mathrm{ILS}C_{1}(n)),n=\bar{1,N}.$$

To measure employee safety awareness, the seven-point scale of Westaby, J.D. and Lee, B.C. (67) can be used, which is also converted into a relative index from 0 to 1.

*At the fifth stage,* the impact on the level of compliance with OH&S requirements of employees is determined, taking into account the influence of employees on each other, due to complex industrial and/or personal ties, they can and should contribute to increasing their awareness in their workplaces:


$$\mathrm{ILS}C_{5}(n)=\mathrm{ILS}C_{4}(n)+\frac{p_{2}w_{n}(1-\mathrm{ILS}C_{4}(n))}{100},n=\bar{1,N},$$

where, the value of $w_{n}$ shows the average influence on awareness of the n -th employee by his/her co-employees:


$$w_{n}=\frac{1}{c_{n}-2}\sum_{\begin{array}{c} i=3:\\ a_{\text{in}}\neq 0\; \end{array}}^{N}a_{\text{in}},n=\bar{1,N}.$$

Here, as in Equation 2, there are no summands that correspond to the manager and the OSS, since the influence of these subjects is taken into account when calculating $\mathrm{ILS}C_{4}(n)$ ; $p_{2}$ —a value indicating by how many per cent (on average) the awareness of the organization’s employees is increased due to the influence of other employees who are related to each other by labor or personal relationships; $a_{\text{in}},i=\bar{3,N},n=\bar{1,N}$ —the degree of influence of enterprise employees on each other. It is convenient to present the results of determining the level of compliance with OH&S requirements of employees for further calculation of the occupational risk level in Table 2.

**Table 2:** Presentation of initial data on the degree of influence of enterprise employees on each other, their personal responsibility and professional risk, compiled based on the results of expert assessments and questionnaires.

Employee	Degree of influence of enterprise employees on each other							Initial occupational risk level
	M	OSS	P_1_	P_2_	P_3_	…	Pn	
M	0	$a_{12}$	$a_{13}$	$a_{14}$	$a_{15}$		$a_{1N}$	$R_{1}$
OSS	$a_{21}$	0	$a_{23}$	$a_{24}$	$a_{25}$		$a_{2N}$	$R_{2}$
P_1_	$a_{31}$	$a_{32}$	0	$a_{34}$	$a_{35}$		$a_{3N}$	$R_{3}$
P_2_	$a_{41}$	$a_{42}$	$a_{43}$	0	$a_{45}$		$a_{4N}$	$R_{4}$
P_3_	$a_{51}$	$a_{52}$	$a_{53}$	$a_{54}$	0		$a_{5N}$	$R_{5}$
…	…	…	…	…	…	…	…	…
Pn	$a_{\mathrm{Nj}}$	$a_{\mathrm{Nj}}$	$a_{\mathrm{Nj}}$	$a_{\mathrm{Nj}}$	$a_{\mathrm{Nj}}$		0	$R_{N}$
Employee serial number	1	2	3	4	5	…	*N*	
Level of compliance with the OHSMS requirements by employees in the absence of influence	$q_{1}$	$q_{2}$	$q_{3}$	$q_{4}$	$q_{5}$		$q_{N}$	

The overall level of compliance with OH&S requirements of employees is calculated by the following equation:


(1) $${\text{GLSC}}_{k}=\sum_{n=1}^{N}{\text{ILSC}}_{k}(n),$$

Where N is the number of employees at the enterprise;

$n,n=\bar{1,N}$ is the employee’s serial number, with n=1 and n=2 corresponding to the Manager (M) and occupational safety specialist (OSS); ${\text{ILSC}}_{k}(n),k=\bar{1.5}$ is individual level of compliance with the OHSMS requirements (Individual Level of Safety Compliance) by n -th employee at k -th stage of the organization’s development, which is measured in conventional units and takes a value from 0 to 1. Evidently, the value of ${\text{GLSC}}_{k}$ at each *k*-th stage ranges from 0 to N . The minimum and maximum in Equation 1 correspond to the lowest and highest levels of occupational safety compliance among all employees, respectively.

The initial (objective) occupational risk level $R_{n}$ for each n -th employee is estimated based on identification of the hazard, hazardous event and severity of consequences, identification of all external and internal hazardous factors, conducting a risk analysis of the hazard from each hazardous factor based on determining the probability of a hazardous event occurring and the degree of severity (37):


(2) $$R_{n}=\sum_{i=1}^{N\_\text{Factors}}B_{\mathrm{ni}}\;T_{\mathrm{ni}},$$

where $R_{n}$ is OH&S risk from hazard for the n -th employee, taking into account all N_Factors of hazardous factors in his/her workplace; ${В}_{\mathrm{ni}}$ is the probability of hazardous event occurring (incident, accident, emergency situations, etc.) under the hazardous factor influence; ${Т}_{\mathrm{ni}}$ is the degree of severity of hazardous event consequences (incident, accident, emergency situations, etc.).

Taking into account the general level of compliance with OH&S requirements of employees, it is possible to specify the level of occupational hazard risk in the workplace of the n-th employee using the formula:


(3) $$\text{Risk}\_\text{leve}l_{n}^{k}=R_{n}+R_{n}(1-{\text{ILSC}}_{k}(n)),n=\bar{1,N},k=\bar{1,5}.$$

Equation 3 reflects the subjective nature of occupational risk for an employee, which increases as the individual level of safety culture decreases.

The overall level of occupational risk at each stage of safety culture development can be assessed using one of the following equations:

- as the maximum:

(4) $${\text{OverallRisk}}_{k}=\max_{\;n=\bar{1,N}}\text{Risk}\_\text{leve}l_{n}^{k},k=\bar{1,5};$$

- as the average:

(5) $${\text{OverallRisk}}_{k}=\frac{1}{N}\sum_{\;n=1}^{N}\text{Risk}\_\text{leve}l_{n}^{k},k=\bar{1,5}.$$

Then we proceed to the next stage, which coincides with the main algorithm for occupational risk management (37), where the OH&S risk level from each hazard is determined as acceptable and unacceptable. If the result is positive, the next stage occurs—documenting occupational risks, followed by the implementation of measures to improve the safety culture level and review the occupational risk level (68–71). In case of an unacceptable risk level, precautionary and protective measures are substantiated (2, 72) with subsequent transition to the stage of analyzing the residual occupational risk level and repeating the specified algorithm.

## 3 Research results

The object of the study is a car service station (CSS) with 14 employees, one manager (M) and one occupational safety specialist (OSS). To identify hazardous factors within the enterprise, the initial occupational risk level $R_{n}$ for each n -th employee, and to assess the degree of their responsibility for safety compliance, five experts from an auditing organization specializing in similar evaluations were involved. These experts met the following criteria: at least 10 years of experience in occupational safety, a higher education degree in occupational safety, and knowledge of ISO 45001 and ISO 19011 standards.

Quantitative variables (scores assigned by experts for each requirement analyzed) were recorded as mean ± standard deviation; qualitative variables were recorded as frequencies and percentages. The Aiken’s V test (73) was used to calculate agreement among experts, thereby quantitatively evaluating the content validity of the questionnaire; resulting values ranged from 0 (no agreement) to 1 (complete agreement among all experts). The 95% confidence intervals were calculated according to Penfield and Giacobbi (74), using Microsoft Excel 365 (Microsoft, Redmond, WA, USA). Validity was confirmed when the lower bound of the interval was >0.7.

Table 3 shows the process of calculating the initial level of compliance with OH&S requirements for each employee ( $q_{n},n=\bar{1,N})$ , based on the results of the occupational safety audit. An example of a fragment of checklists with calculated data is given in the Appendix. Note, this level we calculate in the absence of mutual influence.

**Table 3:** Calculating the initial level of compliance with OH&S requirements for each employee.

Checklist responses	Employee															
	M	OSS	Р_1_	Р_2_	Р_3_	Р_4_	Р_5_	Р_6_	Р_7_	Р_8_	Р_9_	Р_10_	Р_11_	Р_12_	Р_13_	Р_14_
Requirements for organizational context analysis (total of 10)																
Total—Yes	10	10	5	5	6	5	6	5	6	6	5	5	5	5	6	5
Total—No	0	0	5	5	4	5	4	5	4	4	5	5	5	5	4	5
Leadership requirements (total of 18)																
Total—Yes	18	18	6	8	12	6	6	11	6	6	12	4	6	6	12	6
Total—No	0	0	12	10	6	12	12	7	12	12	6	14	12	12	6	12
Requirements for planning (total of 15)																
Total—Yes	15	15	8	5	8	8	8	9	8	8	6	8	8	8	8	8
Total—No	0	0	7	10	7	7	7	6	7	7	9	7	7	7	7	7
Requirements for ensuring OHSMS (total of 18)																
Total—Yes	18	18	9	12	11	9	9	11	9	9	11	12	9	11	11	9
Total—No	0	0	9	6	7	9	9	7	9	9	7	6	9	7	7	9
Requirements for OHSMS operation (total of 18)																
Total—Yes	18	18	12	11	11	12	12	11	12	13	11	10	12	14	11	12
Total—No	0	0	6	7	7	6	6	7	6	5	7	8	6	4	7	6
Requirements for performance evaluation (total of 15)																
Total—Yes	15	15	8	8	9	11	8	9	8	6	9	8	7	12	9	8
Total—No	0	0	7	7	6	4	7	6	7	9	6	7	8	3	6	7
Requirements for OHSMS improvement (total of 11)																
Total—Yes	11	11	7	6	10	6	7	10	7	7	10	7	7	7	10	7
Total—No	0	0	4	5	1	5	4	1	4	4	1	4	4	4	1	4
Total score across all requirements																
Total questions	105															
Total Yes	105	105	55	55	67	57	56	66	56	55	64	54	54	63	67	55
$q_{i}$	1	1	0.5	0.5	0.6	0.5	0.5	0.6	0.5	0.5	0.6	0.5	0.5	0.6	0.6	0.5

The assessment of influence coefficients between employees was carried out based on a structured questionnaire. Selected questions from the developed survey included: Whom do you most frequently consult regarding work-related issues? Whose opinion influences your professional decision-making? Whom do you consider the most authoritative figure in your work environment? Whose feedback or comments prompt you to adjust your behavior or approach to work? With whom do you typically consult before making an important decision? Whose ideas do you usually support during meetings or team discussions? To whom would you entrust an important task if you were unable to complete it yourself?

Each employee rated their colleagues for each question on a scale from 0 to 5, where: 0 indicates no influence; 1–2 indicates weak influence; 3 indicates moderate influence; and 4–5 indicates strong influence. For each colleague, the scores across all questions were summed. The resulting value was normalized by dividing by (5 × the number of questions in the questionnaire). This produced an influence matrix, where rows represent the evaluators and columns represent the individuals being evaluated.

Based on the analysis of the obtained indices, with initial data (Table 4), we computed the individual safety culture level for each employee ${\text{ILSC}}_{k}(n)$ , occupational risk level: individual—by the Equation 3, overall using Equations 4 and 5, according to the above algorithm (Figure 8).

**Table 4:** Input data for the calculation of the level of compliance with OH&S requirements of employees.

Enterprise employees	Degree of influence of enterprise employees on each other																Initial ocupational risk level $R_{n},$ score
	M	OSS	Р_1_	Р_2_	Р_3_	Р_4_	Р_5_	Р_6_	Р_7_	Р_8_	Р_9_	Р_10_	Р_11_	Р_12_	Р_13_	Р_14_	
M	0	0.38	0.46	0.48	0.23	0.13	0.21	0.26	0.55	0.02	0.24	0.26	0.25	0.19	0.27	0.1	4
OSS	0.11	0	0.53	0.22	0.49	0.39	0.22	0.55	0.39	0.4	0.08	0.01	0.35	0.33	0.21	0.27	6
Р_1_	0.16	0	0	0.59	0	0	0	0	0	0	0	0	0	0.55	0	0.3	9
Р_2_	0.59	0	0.21	0	0.38	0	0	0	0	0	0	0	0	0.57	0	0.08	10
Р_3_	0.51	0.52	0.6	0.44	0	0.22	0.23	0.52	0.26	0.09	0.3	0.15	0.31	0.01	0.16	0.46	6
Р_4_	0.31	0	0	0	0.18	0	0.54	0	0	0	0	0	0	0	0	0	3
Р_5_	0	0	0	0	0.43	0.07	0	0.25	0.59	0	0	0	0	0	0	0	5
Р_6_	0	0.3	0	0	0.42	0	0.16	0	0.17	0.25	0	0.15	0	0	0	0	4
Р_7_	0	0	0	0	0	0	0.14	0.12	0	0.51	0	0	0	0	0	0	6
Р_8_	0	0	0	0	0	0	0	0.22	0.1	0	0.15	0.46	0	0	0	0	1
Р_9_	0	0	0	0	0	0	0	0	0	0.49	0	0.09	0.58	0	0	0	5
Р_10_	0	0.42	0	0	0	0	0	0.21	0	0.05	0.29	0	0.22	0.14	0	0	8
Р_11_	0	0	0	0	0	0	0	0	0	0	0.01	0.27	0	0.44	0.21	0	1
Р_12_	0	0	0.4	0.3	0.19	0	0	0	0	0	0	0.53	0.13	0	0.57	0.57	3
Р_13_	0	0	0	0.43	0	0	0	0	0	0	0	0	0.06	0.41	0	0	5
Р_14_	0	0	0.14	0.01	0	0	0	0	0	0	0	0	0	0.14	0	0	7
$q_{i}$	1	1	0.5	0.5	0.6	0.5	0.5	0.6	0.5	0.5	0.6	0.5	0.5	0.6	0.6	0.5	Σ=83

Table 5 presents the calculation results. As can be easily observed, the assessment of the overall level of occupational risk according to Equation 4 can be carried out by considering only those employees whose workplace or type of work is the most hazardous. Equation 5, on the other hand, allows for an evaluation of the general safety situation at the enterprise. Therefore, in subsequent research, we will use the latter.

**Table 5:** Results of calculating the individual and overall level of safety culture and occupational risk.

Enterprise employees	$R_{i}$	${\text{ILSC}}_{k}(n)$					$\text{Risk}\_\text{leve}l_{n}^{k}$				
		k=1	k=2	k=3	k=4	k=5	k=1	k=2	k=3	k=4	k=5
M	4	1	1.0	1.0	1.0	1.0	4.0	4.0	4.0	4.0	4.0
OSS	6	1	1.0	1.0	1.0	1.0	6.0	6.0	6.0	6.0	6.0
Р_1_	9	0.5	0.7	0.7	0.9	0.9	13.5	12.1	11.5	10.1	9.6
Р_2_	10	0.5	0.7	0.8	0.9	1.0	15.0	12.8	12.4	10.7	10.5
Р_3_	6	0.6	0.7	0.8	0.9	0.9	8.4	7.7	7.2	6.5	6.3
Р_4_	3	0.5	0.6	0.6	0.7	0.9	4.5	4.1	4.1	3.8	3.4
Р_5_	5	0.5	0.6	0.7	0.8	0.9	7.5	6.9	6.5	5.9	5.6
Р_6_	4	0.6	0.7	0.8	0.9	0.9	5.6	5.1	5.0	4.5	4.4
Р_7_	6	0.5	0.6	0.7	0.9	0.9	9.0	8.3	7.7	6.8	6.7
Р_8_	1	0.5	0.7	0.8	0.9	0.9	1.5	1.3	1.2	1.1	1.1
Р_9_	5	0.6	0.7	0.8	0.9	0.9	7.0	6.3	6.2	5.6	5.6
Р_10_	8	0.5	0.6	0.7	0.8	0.8	12.0	11.0	10.6	9.7	9.6
Р_11_	1	0.5	0.6	0.7	0.8	0.8	1.5	1.4	1.3	1.2	1.2
Р_12_	3	0.6	0.7	0.7	0.8	0.9	4.2	4.0	3.9	3.7	3.4
Р_13_	5	0.6	0.7	0.8	0.9	0.9	7.0	6.7	6.1	5.5	5.4
Р_14_	7	0.5	0.7	0.8	0.9	0.9	10.5	9.1	8.7	7.6	7.4
Sum	83	${\text{OverallRisk}}_{k}$ as a MAX					15.0	12.8	12.4	10.7	10.5
Average	5,2	${\text{OverallRisk}}_{k}$ as an AVERAGE					7.3	6.7	6.4	5.8	5.6

**Figure 9:**
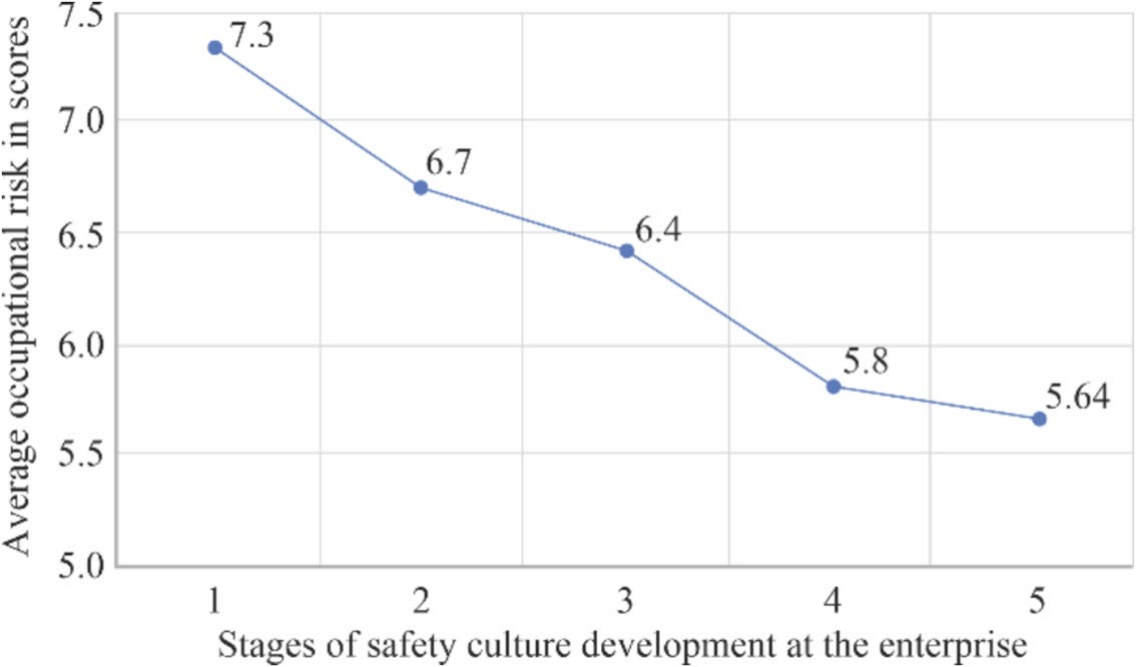
Average occupational risk at the five stages of safety culture development: 1—indifference, 2—response, 3—dependence, 4—independence, 5—interdependence.

The reduction in the average level of occupational risk across all employees of the enterprise, as the level of safety culture and communication improves, is illustrated in Figure 9.

To confirm or refute the hypothesis that the level of safety culture development within an enterprise influences the level of occupational risk—both for individual employees and the organization as a whole—a series of computational experiments was conducted. Input data for 16 of these experiments are presented in Tables 6, 7. Table 6 contains the coefficients of interpersonal influence among employees $a_{\text{in}},i=\bar{1,N},n=\bar{1,N}$ , which have been slightly modified compared to those presented in Table 4. Table 7 provides data that vary across experiments, including the initial levels *q_n_* of compliance with the OHSMS requirements, as well as the percentage increase in safety awareness attributed to interpersonal influence $p_{1}$ and $p_{2}$ .

**Table 6:** Degree of influence of enterprise employees on each other for the experiments.

Enterprise employees	Degree of influence of enterprise employees on each other															
	M	OSS	Р_1_	Р_2_	Р_3_	Р_4_	Р_5_	Р_6_	Р_7_	Р_8_	Р_9_	Р_10_	Р_11_	Р_12_	Р_13_	Р_14_
M	0	0.38	0.46	0.48	0.63	0.13	0.41	0.26	0.55	0.72	0.24	0.26	0.25	0.19	0.67	0.5
OSS	0.61	0	0.53	0.72	0.49	0.39	0.42	0.55	0.39	0.6	0.58	0.41	0.35	0.33	0.21	0.67
Р_1_	0.36	0	0	0.59	0	0	0	0	0	0	0	0	0	0.55	0	0.6
Р_2_	0.59	0	0.71	0	0.38	0	0	0	0	0	0	0	0	0.57	0	0.08
Р_3_	0.51	0.52	0.6	0.44	0	0.82	0.23	0.52	0.26	0.09	0.3	0.15	0.31	0.71	0.16	0.46
Р_4_	0.31	0	0	0	0.18	0	0.54	0	0	0	0	0	0	0	0	0
Р_5_	0	0	0	0	0.43	0.57	0	0.25	0.59	0	0	0	0	0	0	0
Р_6_	0	0.7	0	0	0.42	0	0.56	0	0.51	0.25	0	0.55	0	0	0	0
Р_7_	0	0	0	0	0	0	0.14	0.72	0	0.51	0	0	0	0	0	0
Р_8_	0	0	0	0	0	0	0	0.22	0.7	0	0.15	0.46	0	0	0	0
Р_9_	0	0	0	0	0	0	0	0	0	0.49	0	0.09	0.58	0	0	0
Р_10_	0	0.42	0	0	0	0	0	0.61	0	0.65	0.29	0	0.82	0.49	0	0
Р_11_	0	0	0	0	0	0	0	0	0	0	0.81	0.27	0	0.44	0.21	0
Р_12_	0	0	0.8	0.6	0.43	0	0	0	0	0	0	0.53	0.13	0	0.57	0.57
Р_13_	0	0	0	0.43	0	0	0	0	0	0	0	0	0.86	0.41	0	0
Р_14_	0	0	0.61	0.51	0	0	0	0	0	0	0	0	0	0.66	0	0

**Table 7:** Initial data for the experiments of calculating the occupational risk level taking into account the overall level of compliance of all employees with OH&S requirements of employees.

Experiment No	Level of compliance of all employees with OH&S requirements of employees taking into account their influence on each other, $q_{i}$																Increase in awarenes s, %	
	M	OSS	Р_1_	Р_2_	Р_3_	Р_4_	Р_5_	Р_6_	Р_7_	Р_8_	Р_9_	Р_10_	Р_11_	Р_12_	Р_13_	Р_14_	$p_{1}$	$p_{2}$
1	1	1	0	0	0	0	0	0	0	0	0	0	0	0	0	0	60	70
2	1	0	0	0	0	0	0	0	0	0	0	0	0	0	0	0	60	70
3	1	0	0.7	0.8	0.2	0.7	0.6	0.7	0.6	0.8	0.7	0.6	0.8	1	0.1	0.4	60	70
4	1	0.6	0.7	0.8	0.2	0.7	0.6	0.7	0.6	0.8	0.7	0.6	0.8	1	0.1	0.4	60	70
5	1	1	0.7	0.8	0.2	0.7	0.6	0.7	0.6	0.8	0.7	0.6	0.8	1	0.1	0.4	60	70
6	1	1	0.7	0.8	0.8	0.7	0.6	0.7	0.6	0.8	0.7	0.6	0.8	1	0.7	0.4	60	70
7	0.7	1	0.7	0.8	0.8	0.7	0.6	0.7	0.6	0.8	0.7	0.6	0.8	1	0.7	0.9	60	70
8	1	1	1	1	1	1	1	1	1	1	1	1	1	1	1	1	60	100
9	0.7	1	0.7	0.8	0.8	0.7	0.6	0.7	0.6	0.8	0.7	0.6	0.8	1	0.7	0.9	80	90
10	0.7	1	0.7	0.8	0.8	0.7	0.6	0.7	0.6	0.8	0.7	0.6	0.8	1	0.7	0.9	80	95
11	0.8	0.9	0.8	0.8	0.7	0.7	0.6	0.7	0.6	0.8	0.7	0.6	0.8	0.9	0.7	0.9	80	95
12	0.7	0.7	0.7	0.5	0.3	0.7	0.6	0.9	0.6	0.3	0.7	0.6	0.8	0.8	0.7	0.4	80	90
13	0.6	0.5	0.7	0.5	0.5	0.7	0.6	0.7	0.6	0.3	0.7	0.6	0.8	0.7	0.7	0.8	80	95
14	0.8	0.3	0.3	0.3	0.3	0.6	0.3	0.6	0.5	0.3	0.7	0.6	0.8	0.7	0.7	0.8	80	95
15	0.9	0.2	0.5	0.5	0.5	0.5	0.5	0.5	0.5	0.5	0.5	0.5	0.5	0.5	0.5	0.8	80	95
16	0.9	0.9	0.7	0.8	0.9	0.7	0.6	0.7	0.6	0.8	0.7	0.6	0.8	0.9	0.7	0.9	80	95

The results of the computational experiments are presented in Table 8. Lines 2 and 8 in Tables 7, 8 represent the worst and best hypothetical situations for the enterprise’s safety culture. The worst option is that the enterprise does not have an occupational safety specialist position on staff, and all employees, except the manager, neglect their duties in terms of compliance with safety requirements. This situation is considered in order to show that, with equations given above, the minimum possible safety culture level at the enterprise is 1, while the occupational risk level doubles (in the absence of influential relationships among employees). In the best case, all employees of the enterprise at the highest level carefully comply with the OHSMS requirements, which means that the overall safety culture level takes a value equal to the number of employees (in percentage equivalent—100%) and the occupational risk level is equal to the maximum of the objective ones calculated for each workplace: $\max_{n}R_{n}=10$ .

**Table 8:** Results of calculating the occupational risk level at each stage of the organization’s development, taking into account the increase in employee awareness.

Experiment No	${\text{OverallRisk}}_{k}$ calculated by Equation 5					Increasing the occupational risk level ${\text{OverallRisk}}_{k}$ at k− th stage of safety culture development, *per cent*				
	k=0	k=1	k=2	k=3	k=4	k=0	k=1	k=2	k=3	k=4
1	9.8	8.4	7.8	6.5	6.1	88.0	61.5	50.3	25.2	18.5
2	10.1	10.1	8.9	8.0	7.2	95.2	95.2	71.0	54.9	39.4
3	7.4	7.4	6.8	6.4	6.1	43.1	43.1	31.1	23.2	16.7
4	7.2	6.9	6.5	6.0	5.8	38.8	32.7	24.5	15.0	11.1
5	7.1	6.5	6.2	5.7	5.6	35.9	25.7	20.1	9.6	7.3
6	6.6	6.2	6.0	5.6	5.5	28.0	19.5	15.9	7.8	5.9
7	6.5	6.1	6.0	5.7	5.6	25.2	17.9	15.6	9.3	7.0
8	5.2	5.2	5.2	5.2	5.2	0.0	0.0	0.0	0.0	0.0
9	6.5	6.0	5.8	5.7	5.50	25.2	15.4	12.5	9.3	6.4
10	6.5	6.0	5.8	5.7	5.5	25.2	15.4	12.5	9.3	6.2
11	6.5	6.1	5.9	5.7	5.5	25.4	16.9	13.0	9.9	6.6
12	7.2	6.7	6.3	6.1	5.8	39.2	28.2	22.0	17.7	11.2
13	7.3	6.9	6.6	6.4	5.9	41.0	33.1	26.8	23.3	14.4
14	7.8	7.5	6.9	6.6	6.0	49.6	44.1	32.3	28.0	16.5
15	7.8	7.6	6.9	6.7	6.1	50.2	46.7	32.9	28.6	17.5
16	6.5	6.0	5.8	5.7	5.5	24.6	16.4	12.2	9.1	6.0
Increasing the occupational risk level average for all experiments:						39.7	32.0	24.6	17.5	11.9

In the remaining experiments, it is possible to observe the influence of the level of compliance with OH&S requirements of employees on the increase in the occupational risk level:

(1) At each stage of the enterprise’s development with a change in such parameters as the initial level of compliance with the OHSMS requirements, percentages of increased awareness $p_{1}$ and $p_{2}$ of the employee due to the influence of the opinions of his/her colleagues (according to lines of Table 8);
(2) When moving from stage to stage (according to columns of Table 8).

Thus, Table 8 shows the results of calculating the average occupational risk level for all employees and the percentage by which this value differs from the initial one (which equals 5.2) at each stage of the enterprise’s development. Calculations are made using the above data and the percentages of increased awareness $p_{1}$ and $p_{2}$ of employees indicated in the second and third columns due to the influence of the opinions of his/her co-employees.

**Figure 10:**
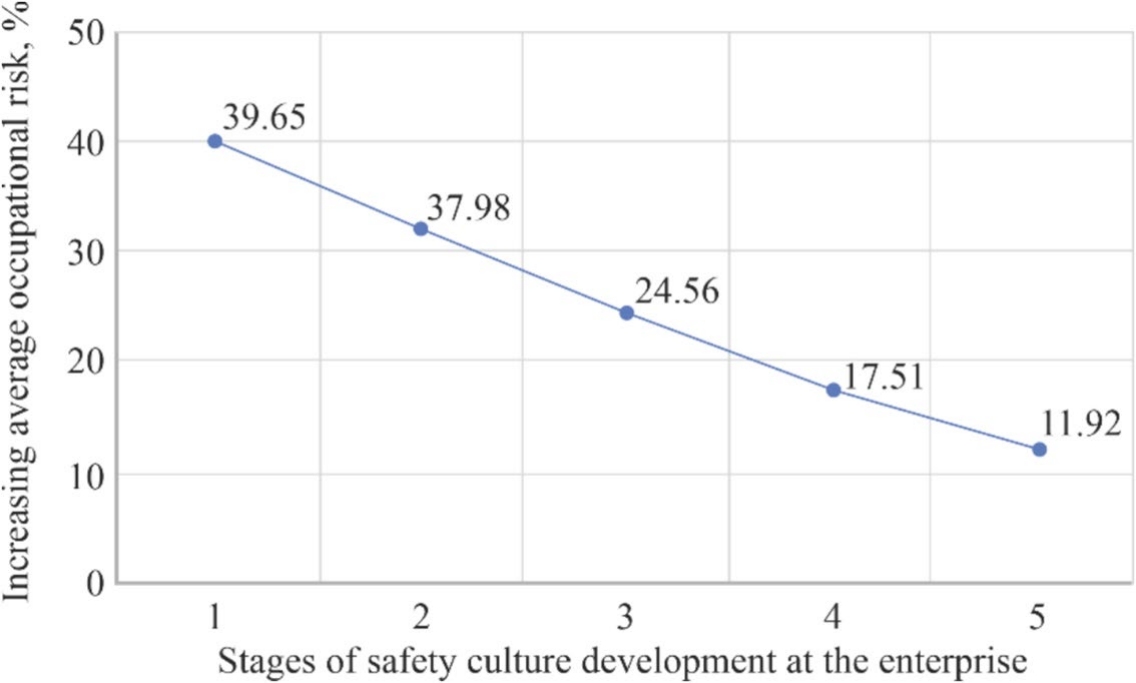
Reduction (in percentage) of the occupational risk value by improving the safety culture at the enterprise in average for all employees and all computing experiments.

The last line of Table 8 provides data on the percentages by which, the average occupational risk level at each stage of the enterprise development with an appropriate safety culture level has increased for all experiments. Figure 10 presents this information in the form of a dependency graph of increasing value (in percentage equivalent) of the average occupational risk (y-axis) on the safety culture level, which corresponds to the stage of the enterprise’s development (x-axis).

Obviously, the average occupational risk level decreases with an increase in the authority of occupational safety specialists, the involvement of administrative resources from management, the emergence of self-awareness among employees to comply with requirements and the influence of mutual assistance among employees. But at the stage of enterprise formation, when labour and personal relationships between employees have not yet been established, the occupational risk at the enterprise is significantly greater than that calculated on condition of 100% compliance of all employees with safety requirements.

## 4 Discussion

To manage occupational risks, it is necessary to consider the safety culture level in the organization, which will further improve the effectiveness of the occupational health and safety management system by identifying the influence of relationships among employees regarding their commitment to compliance with OH&S requirements of employees. This conclusion is based on the analysis of the obtained correlation between the occupational risk level of incident occurrence for each level of safety culture development and the change in such parameters as the initial individual level of compliance with OH&S requirements of employees, the increase in the employee awareness to comply with OH&S requirements of employees, the presence of mutual assistance between colleagues in compliance with OH&S requirements of employees. In this case, it is known that taking care of one’s own safety while performing production tasks on the basis of personal initiative, proactive attitude, teamwork based on common values significantly reduces the level of injuries in the organization compared to using only natural survival instincts of an employee or manager’s control of occupational safety and health protection (75, 76), which confirms the conclusion made.

In addition, the existence of a correlation between the level of safety culture and the number of incidents is supported by the analysis of the literature (29–32). The failure to comply with occupational health and safety (OHS) requirements is a major cause of workplace injuries. Therefore, we propose a quantitative approach to assess the impact of non-compliance on the level of occupational risk. According to various studies, the risk level may increase by up to 40% in cases of complete disregard for OHS regulations, and by up to 12% when appropriate influence is exerted by unit managers, safety specialists, and the employees themselves on each other. The degree of compliance with OHS requirements is one of the key factors that can significantly improve the accuracy of risk assessment. Both our findings and those of other authors (34, 35) suggest that increasing employee awareness of the importance of safety compliance contributes to a reduction in occupational risk. Employees’ awareness and motivation to follow safety rules are influenced by the authority and safety attitudes of senior management (41, 46), the influence of informal leaders within specific units (47), and the behavior and commitment of occupational safety professionals in fulfilling their responsibilities (48). Other critical factors include safety training, implementation of feedback mechanisms, monitoring and reporting systems, and related measures.

When forming the managers’ attitude to occupational health and safety under different models, it is taken into account that the effectiveness of the occupational health and safety management system largely depends on how managers, occupational safety specialists and employees understand, assess and take into account in their actions the importance to comply with OH&S requirements of employees, determined precisely by the safety culture level (77, 78). Hence, there is a need to take into account the level of development of the company’s “occupational safety culture” as an additional characteristic to clarify the hazardous factors influencing the occupational risk level. This allows us to state that a significant change in the level of compliance with OH&S requirements of employees leads to a decrease in the occupational risk level due to the increased interest of managers and employees’ awareness of the need and importance to comply with OH&S requirements of employees. At the same time, the proposal to determine the safety culture level based on the indicator of employees’ influence on the level of compliance with requirements of employees allows to characterize the effectiveness of the management system functioning by setting appropriate relative indices. This possibility of application is confirmed by research (40), where the authors indicate that the most influential indicators characterizing the level of occupational safety culture should include: safety awareness of the participants of the production process and attitude of managers and employees to the safety issues. Indirectly indicating that the specified four indicators characterize the level of safety culture, researchers note that by changing the attitude of managers towards occupational safety, the awareness of the need to comply with occupational safety rules by other employees significantly increases (69, 79, 80). As a result, this will affect the probability of a hazardous event occurring by reducing the number of hazardous factors, and thus the occupational risk level, thereby increasing the efficiency of the OHSMS as a whole. Based on these indicators, it is possible to determine the influence of employee indifference on the emergence of three components: negligence, incompetence (lack of appropriate training) and the presence of selfish motives, which generally characterizes the safety culture level (61, 63). From here, it is possible to predict the occurrence of hazardous situations, either with a specific employee or the organization as a whole, due to the presence of a significant number of non-compliance with requirements. A conclusion drawn from the Bradley curve shows that with individual responsibility for occupational safety at low safety culture levels (response, supervision), the injury rate is significantly higher than at levels with mutual assistance among employees (81, 82). In addition, a number of scientific empirical studies have drawn similar conclusions regarding the dependence of the injury rate on the safety culture level. In particular, Chen and Yang show an existing relationship between an increase in the injury risk index, based on the assessment of hazardous activities and hazardous conditions (83). A similar idea was implemented in (84) for predicting the injury rate.

Also, the overall level of compliance with OH&S requirements of employees, as a result, makes it possible to assess the effectiveness of occupational safety measures by adjusting the occupational risk level from the totality of hazardous factors (85, 86).

It is recommended to managers of organizations to try to shape safe behavior in their employees by their own example and create an occupational health and safety management system, which includes a number of basic components:

(1) Actual values of the enterprise, including occupational safety and health protection of employees;
(2) Policy in the field of occupational safety and health protection of employees;
(3) Principles of ensuring occupational safety and health protection of employees, as well as the management system;
(4) Formation of commitment of employees to occupational safety and health protection issues;
(5) Personnel behavioral response system;
(6) Training and motivation of personnel to improve occupational safety and health protection of employees;
(7) Effective occupational risk management procedures that are put into practice and have a significant impact on reducing the loss of life and health of employees;
(8) Occupational safety and health management system of employees;
(9) Management of non-conformities and incidents in the field of occupational safety and health protection of employees;
(10) A model for the development of both the enterprise itself and the “occupational safety culture” of its employees.

The limitations of the present study include the complexity of analyzing interpersonal influences among a large number of employees in large-scale organizations, particularly in the context of their attitudes toward compliance with occupational health and safety (OHS) requirements. Additionally, the obtained risk values reflect only general trends in risk reduction associated with different levels of safety culture. The specific risk values apply solely to the particular enterprise under study, although they were calculated under varying initial conditions. This research was conducted primarily to demonstrate the sequence of calculations as an example for practitioners who may be interested in applying this methodology. Furthermore, when assessing employees’ attitudes toward OHS compliance, the BFI-10 form (56, 87) was used, which evaluates only five personality traits. Future studies may benefit from applying alternative methods for assessing initial data, including the consideration of potential negative peer influence and other relevant factors.

## 5 Conclusion

A process for assessing and managing occupational risks has been developed, taking into account the level of compliance with OH&S requirements of employees, based on the five levels of safety culture that correspond to the Bradley curve: indifference, response, dependence, independence, interdependence which makes it possible to determine the attitude of the manager, employee and occupational safety specialist to compliance with occupational safety rules.

It is proposed to determine the occupational risk level taking into account the level of compliance with OH&S requirements of employees, which represents the sum of the levels of compliance with requirements by each employee considering the influence of managers and occupational safety specialists, the influence of employees themselves and self-awareness on the degree of compliance with OH&S requirements of employees. The occupational risk level can be determined using the reports on supervision, audit, questionnaires developed based on the basic national requirements for the occupational health and safety management system for employees, etc.

A matrix of a correlation between the influence of the manager, occupational safety specialist and employees on the level of compliance with OH&S requirements of employees and the levels of safety culture development is proposed, which allows determining the attitude of employees to compliance with occupational safety requirements, thereby reducing the probability of a hazardous event occurring or its severity due to the reduction in the number and influence of hazardous factors, and consequently the occupational risk level.

The dependence has been revealed of the occupational risk for each level of safety culture development on changes in such parameters as the initial individual level of compliance with OH&S requirements of employees, the increase in the employee awareness to comply with OH&S requirements, the presence of mutual assistance between colleagues in compliance with OH&S requirements of employees.

It has been found that a significant change in the level of compliance with OH&S requirements of employees leads to a decrease in the occupational risk level to an acceptable level due to the increased interest of managers, occupational safety specialist and employees’ awareness of the need and importance to comply by everyone with OH&S requirements of employees.

The dependence has been determined of the occupational risk level at each “safety culture” level on the initial level of compliance with OH&S requirements of employees and increase in the employee awareness to comply with occupational safety rules due to the influence of employees, occupational safety specialists and managers, as well as self-awareness of employees and their mutual assistance in compliance with OH&S requirements of employees.

## Data Availability

The original contributions presented in the study are included in the article/Supplementary material, further inquiries can be directed to the corresponding author.
