# Supplementary material for: Influence of occupational safety culture on the occupational risk level in the organization
Source: Front Public Health. 2025 Jun 25;13:1595869. doi: 10.3389/fpubh.2025.1595869 (PMC12237939; doi:10.3389/fpubh.2025.1595869)
Supplement: Supplementary file 1 [file Table_1.docx]

Appendix

Fragment of the internal audit checklist for determining the requirement fulfillment coefficient in the workplace unit in accordance with ISO 45001 standard

| **№** | **Requirements defined in the enterprise’s OH&S management system (OHSMS) in accordance with ISO 45001** | | **Requirement Code** | **Requirement Fulfillment** | | | **Remarks** |  |
| --- | --- | --- | --- | --- | --- | --- | --- | --- |
|  |  |  |  | **Yes «+»** | **No «-»** |  | |  |
| - **Group 1. Requirements for analysis of the organizational context (*i* = 1)**   (1 – group number of requirements in section 4, *j –* requirement number in group 1 of section 4) | | | | | | | | |
| 1. | Are external factors at the workplace identified that influence the ability to achieve the intended outcome? | | *В_11_* |  |  |  | |  |
| 2 | Are internal factors at the workplace identified that influence the ability to achieve the intended outcome? | | *В_12_* |  |  |  | |  |
| 3 | Are all interested parties that influence the OH&S management system identified? | | *В_13_* |  |  |  | |  |
| 4 | Are the needs and expectations of interested parties identified? | | *В_14_* |  |  |  | |  |
| 5 | Is it determined which of these needs and expectations may become legal requirements? | | *В_15_* |  |  |  | |  |
| 6 | Is the scope of the OH&S management system established? | | *В_16_* |  |  |  | |  |
| 7 | Are all types of activities of the unit considered when determining the scope of application? | | *В_17_* |  |  |  | |  |
| 8 | Are interested parties, their needs, and expectations taken into account when defining the scope of application? | | *В_18_* |  |  |  | |  |
| 9 | Is the scope of the OH&S management system documented? | | *В_19_* |  |  |  | |  |
| 10 | Is information about the scope of application available to interested parties? | | *В_110_* |  |  |  | |  |
| … | … | | *…* | … | … |  | |  |
| j |  | | *В_ij_* | … | … |  | |  |
| … | … | | *…* | … | … |  | |  |
| n_1_ | … | | *В_1n1_* | … | … |  | |  |
| Total number of requirements: | | | *n_1_ = n_1_^+^ + n_1_^-^* | *n_1_^+^* | *n_1_^-^* |  | |  |
| Requirement fulfillment coefficient: | | | *Кв_1_ = n1^+^/ n_1_* | *Кв_1_* | *-* |  | |  |
| - **Group 2. Requirements for leadership in the OH&S management system (*i* = 2)**   **(**2 – group number of requirements in section 5, *j* – requirement number in group 2 of section 5) | | | | | | | | |
| 1. | Does the unit management demonstrate leadership and commitment to the OH&S management system? | | *В_21_* |  |  |  | |  |
| 2 | Does the management provide adequate resources for the functioning of the OH&S management system? | | *В_22_* |  |  |  | |  |
| 3 | Are responsibilities and authorities regarding occupational health and safety defined? | | *В_23_* |  |  |  | |  |
| 4 | Does the management ensure the integration of OH&S management system requirements into business processes? | | *В_24_* |  |  |  | |  |
| 5 | Does the management support a culture that promotes workplace safety? | | *В_25_* |  |  |  | |  |
| 6 | Do managers discuss issues related to improving occupational safety with employees? | | *В_26_* |  |  |  | |  |
| 7 | Do managers personally participate in safety activities, monitor, and provide assistance? | | *В_27_* |  |  |  | |  |
| 8 | Has the OH&S policy been developed and approved? | | *В_28_* |  |  |  | |  |
| 9 | Does the policy include a commitment to preventing injuries and illnesses? | | *В_29_* |  |  |  | |  |
| 10 | Does the policy include a commitment to consultation and participation of workers in OH&S matters? | | *В_210_* |  |  |  | |  |
| 11 | Is the policy documented and available to interested parties? | | *В_211_* |  |  |  | |  |
| 12 | Has the policy been communicated to employees? | | *В_212_* |  |  |  | |  |
| 13 | Is the policy regularly reviewed and updated? | | *В_213_* |  |  |  | |  |
| 14 | Is it ensured that employees are aware of their roles and responsibilities within the OH&S management system? | | *В_214_* |  |  |  | |  |
| 15 | Is employee participation in OH&S matters ensured? | | *В_215_* |  |  |  | |  |
| 16 | Are employees provided with opportunities for consultation on OH&S issues? | | *В_216_* |  |  |  | |  |
| 17 | Are employees informed of their rights and obligations regarding OH&S? | | *В_217_* |  |  |  | |  |
| 18 | Does the organization promote employee involvement in hazard identification and proposals for improvement of the OH&S management system? | | *В_218_* |  |  |  | |  |
| … | … | | *…* | …. | …. |  | |  |
| j | … | | *В_ij_* | … | … |  | |  |
| … | … | | *…* | … | … |  | |  |
| n_2_ | … | | *В_2n2_* | … | … |  | |  |
| Total number of requirements: | | | *n_2_ = n_2_^+^ + n_2_^-^* | *n_2_^+^* | *n_2_^-^* |  | |  |
| Requirement fulfillment coefficient: | | | *Кв_1_ = n2^+^/ n_2_* | *Кв_2_* | *-* |  | |  |
| - **Group 3. Requirements for planning in the OH&S management system (*i*= 3)**   **(**3 – group number of requirements in section 6*, j* – requirement number in group 3 of section 6) | | | | | | | | |
| 1. | Are risks and opportunities for the OH&S management system and its intended outcomes identified? | | *В_31_* |  |  |  | |  |
| 2 | Are routine and non-routine activities and situations, along with risks associated with workplace infrastructure, analyzed? | | *В_32_* |  |  |  | |  |
| 3 | Are external and internal factors that influence the OH&S management system considered during planning? | | *В_33_* |  |  |  | |  |
| 4 | Are mechanisms implemented to eliminate or minimize OH&S risks? | | *В_34_* |  |  |  | |  |
| 5 | Are opportunities for improving the OH&S management system utilized? | | *В_35_* |  |  |  | |  |
| 6 | Are OH&S objectives established at the unit level? | | *В_36_* |  |  |  | |  |
| 7 | Do the objectives align with the OH&S policy? | | *В_37_* |  |  |  | |  |
| 8 | Are the results of risk and opportunity assessments considered when setting objectives? | | *В_38_* |  |  |  | |  |
| 9 | Are indicators defined for evaluating the achievement of objectives? | | *В_39_* |  |  |  | |  |
| 10 | Are action plans developed to achieve OH&S objectives? | | *В_310_* |  |  |  | |  |
| 11 | Are resources, responsible persons, and deadlines specified in the plans? | | *В_311_* |  |  |  | |  |
| 12 | Are processes defined for planning changes that may affect the OH&S management system? | | *В_312_* |  |  |  | |  |
| 13 | Are the potential consequences of changes evaluated? | | *В_313_* |  |  |  | |  |
| 14 | Are measures taken to prevent negative consequences of changes? | | *В_314_* |  |  |  | |  |
| 15 | Is employee participation ensured in planning changes? | | *В_315_* |  |  |  | |  |
| … | … | | *…* | …. | …. |  | |  |
| j | … | | *В_ij_* | … | … |  | |  |
| … | … | | *…* | … | … |  | |  |
| n_3_ | … | | *В_3n3_* | … | … |  | |  |
| Total number of requirements: | | | *n_3_ = n_3_^+^ + n_3_^-^* | *n_3_^+^* | *n_3_^-^* |  | |  |
| Requirement fulfillment coefficient: | | | *Кв_3_ = n_3_^+^/ n_3_* | *Кв_3_* | *-* |  | |  |
| - **Group 4. Requirements for support in the OH&S management system (*i* = 4)**   **(**4 – group number of requirements in section, *j* – requirement number in group 4 of section 7) | | | | | | | | |
| 1. | Does the unit plan and implement processes that ensure compliance with OH&S requirements? | | *В_41_* |  |  |  | |  |
| 2 | Does the unit develop, implement, and maintain processes to eliminate hazards and risks? | | *В_42_* |  |  |  | |  |
| 3 | Are the necessary resources provided for implementing, maintaining, and improving the OH&S management system? | | *В_43_* |  |  |  | |  |
| 4 | Are resource needs considered when planning OH&S activities? | | *В_44_* |  |  |  | |  |
| 5 | Are financial, technical, and human resources provided for the functioning of the OH&S management system? | | *В_45_* |  |  |  | |  |
| 6 | Are the required competencies of employees influencing the effectiveness of the OH&S management system determined? | | *В_46_* |  |  |  | |  |
| 7 | Is training and professional development of employees ensured to meet OH&S management system requirements? | | *В_47_* |  |  |  | |  |
| 8 | Is the effectiveness of training activities evaluated? | | *В_48_* |  |  |  | |  |
| 9 | Are the results of employee training documented? | | *В_49_* |  |  |  | |  |
| 10 | Are employees aware of their roles within the OH&S management system? | | *В_410_* |  |  |  | |  |
| 11 | Are employees aware of the consequences of non-compliance with OH&S management system requirements? | | *В_411_* |  |  |  | |  |
| 12 | Are internal and external communications related to the OH&S management system defined? | | *В_412_* |  |  |  | |  |
| 13 | Is effective information exchange between employees and management regarding occupational health and safety ensured? | | *В_413_* |  |  |  | |  |
| 14 | Are the needs of interested parties considered when planning communications? | | *В_414_* |  |  |  | |  |
| 15 | Is documentation developed regarding the functioning of the OH&S management system? | | *В_415_* |  |  |  | |  |
| 16 | Is this documentation available to interested parties? | | *В_416_* |  |  |  | |  |
| 17 | Is such documentation regularly updated? | | *В_417_* |  |  |  | |  |
| 18 | Is the protection of confidential information related to the OH&S management system ensured? | | *В_418_* |  |  |  | |  |
| … | … | | *…* | …. | …. |  | |  |
| J | … | | *В_ij_* | … | … |  | |  |
| … | … | | *…* | … | … |  | |  |
| n_4_ | … | | *В_4n4_* | … | … |  | |  |
| Total number of requirements: | | | *n_4_ = n_4_^+^ + n_4_^-^* | *n_4_^+^* | *n_4_^-^* |  | |  |
| Requirement fulfillment coefficient: | | | *Кв_4_ = n_4_^+^/ n_4_* | *Кв_4_* | *-* |  | |  |
| - **Group 5. Requirements for operation in the OH&S management system (*i*= 5)**   **(**5 – group number of requirements in section 8, *j –* requirement number in group 5 of section 8) | | | | | | | | |
| 1. | Has a procedure been developed for monitoring, measuring, analyzing, and evaluating the effectiveness of production equipment safety systems? | | *В_51_* |  |  |  | |  |
| 2 | Are compliance criteria with OH&S requirements established for processes within the unit? | | *В_52_* |  |  |  | |  |
| 3 | Is control exercised over processes according to these criteria? | | *В_53_* |  |  |  | |  |
| 4 | Are processes carried out as planned? | | *В_54_* |  |  |  | |  |
| 5 | Have all hazards been eliminated, if possible? | | *В_55_* |  |  |  | |  |
| 6 | Have processes and equipment been substituted with less hazardous alternatives, if possible? | | *В_56_* |  |  |  | |  |
| 7 | Have technical measures been applied to reduce risks? | | *В_57_* |  |  |  | |  |
| 8 | Have administrative control measures been applied to reduce risks? | | *В_58_* |  |  |  | |  |
| 9 | Have personal and collective protective equipment been used to reduce risks? | | *В_59_* |  |  |  | |  |
| 10 | Are processes defined for managing changes that may affect the OH&S management system? | | *В_510_* |  |  |  | |  |
| 11 | Are procurement requirements related to the OH&S management system defined? | | *В_511_* |  |  |  | |  |
| 12 | Are suppliers and contractors evaluated for compliance with OH&S requirements? | | *В_512_* |  |  |  | |  |
| 13 | Is communication with suppliers and contractors regarding OH&S management system requirements ensured? | | *В_513_* |  |  |  | |  |
| 14 | Is compliance with OH&S requirements monitored during contractors' work performance? | | *В_514_* |  |  |  | |  |
| 15 | Are potential emergency situations identified? | | *В_515_* |  |  |  | |  |
| 16 | Are action plans developed for emergency situations? | | *В_516_* |  |  |  | |  |
| 17 | Are drills or training sessions conducted for emergency response? | | *В_517_* |  |  |  | |  |
| 18 | Are action plans reviewed and updated after emergencies or drills? | | *В_518_* |  |  |  | |  |
| … | … | | *…* | …. | …. |  | |  |
| J | … | | *В_ij_* | … | … |  | |  |
| … | … | | *…* | … | … |  | |  |
| n_5_ | … | | *В_5n5_* | … | … |  | |  |
| Total number of requirements: | | | *n_5_ = n_5_^+^ + n_5_^-^* | *n_5_^+^* | *n_5_^-^* |  | |  |
| Requirement fulfillment coefficient: | | | *Кв_5_ = n_5_^+^/ n_5_* | *Кв_5_* | *-* |  | |  |
| - **Group 6. Requirements for performance evaluation in the OH&S management system(*i* = 6)**   **(**6 – group number of requirements in section 9, *j* – requirement number in group 6 of section 9) | | | | | | | | |
| 1. | | Are criteria defined for evaluating the effectiveness of the OH&S management system? | *В_61_* |  |  |  | |  |
| 2 | | Are regular inspections and evaluations of compliance with OH&S requirements conducted? | *В_62_* |  |  |  | |  |
| 3 | | Are tools used for monitoring and measuring the performance of the OH&S management system? | *В_63_* |  |  |  | |  |
| 4 | | Is data regarding incidents, accidents, and other OH&S-related events analyzed? | *В_64_* |  |  |  | |  |
| 5 | | Is the effectiveness of risk mitigation measures evaluated? | *В_65_* |  |  |  | |  |
| 6 | | Are evaluations of compliance with legal and other requirements conducted? | *В_66_* |  |  |  | |  |
| 7 | | Are internal audits of the OH&S management system planned and conducted? | *В_67_* |  |  |  | |  |
| 8 | | Are criteria and frequencies for conducting internal audits defined? | *В_68_* |  |  |  | |  |
| 9 | | Is the independence and objectivity of auditors ensured? | *В_69_* |  |  |  | |  |
| 10 | | Are the results of internal audits documented? | *В_610_* |  |  |  | |  |
| 11 | | Are corrective actions taken based on audit results? | *В_611_* |  |  |  | |  |
| 12 | | Are regular management reviews of the OH&S management system conducted? | *В_612_* |  |  |  | |  |
| 13 | | Are the results of monitoring, audits, and evaluations considered during management reviews? | *В_613_* |  |  |  | |  |
| 14 | | Are priorities and directions for improvement of the OH&S management system determined during management reviews? | *В_614_* |  |  |  | |  |
| 15 | | Are decisions and actions taken during management reviews documented? | *В_615_* |  |  |  | |  |
| … | | … | *…* | …. | …. |  | |  |
| J | | … | *В_ij_* | … | … |  | |  |
| … | | … | *…* | … | … |  | |  |
| n_6_ | | … | *В_6n6_* | … | … |  | |  |
| Total number of requirements: | | | *n_6_ = n_6_^+^ + n_6_^-^* | *n_6_^+^* | *n_6_^-^* |  | |  |
| Requirement fulfillment coefficient: | | | *Кв_6_ = n_6_^+^/ n_6_* | *Кв_6_* | *-* |  | |  |
| - **Group 7. Requirements for improvement in the OH&S management system (*i* = 7)**   **(**7 – group number of requirements in section 10, *j* – requirement number in group 7 of section 10) | | | | | | | | |
| 1. | | Are opportunities for improving the OH&S management system identified within the unit? | *В_71_* |  |  |  | |  |
| 2 | | Are actions implemented to improve the OH&S management system? | *В_72_* |  |  |  | |  |
| 3 | | Are processes for identifying and managing incidents and nonconformities developed, implemented, and maintained within the unit? | *В_73_* |  |  |  | |  |
| 4 | | Are immediate actions taken to eliminate the consequences of incidents and nonconformities? | *В_74_* |  |  |  | |  |
| 5 | | Are the causes of incidents and nonconformities analyzed? | *В_75_* |  |  |  | |  |
| 6 | | Are corrective actions implemented to prevent the recurrence of similar incidents? | *В_76_* |  |  |  | |  |
| 7 | | Is the effectiveness of corrective actions evaluated? | *В_77_* |  |  |  | |  |
| 8 | | Are incidents and nonconformities, along with the actions taken, documented? | *В_78_* |  |  |  | |  |
| 9 | | Are audit results, evaluations, and data analysis used to identify opportunities for improvement? | *В_79_* |  |  |  | |  |
| 10 | | Are measures to improve the effectiveness of the OH&S management system implemented based on these results? | *В_710_* |  |  |  | |  |
| 11 | | Are improvement objectives and plans regularly reviewed and updated? | *В_711_* |  |  |  | |  |
| … | | … | *…* | …. | …. |  | |  |
| j | | … | *В_ij_* | … | … |  | |  |
| … | | … | *…* | … | … |  | |  |
| n_7_ | | … | *В_7n7_* | … | … |  | |  |
| Total number of requirements: | | | *n_7_= n_7_^+^ + n_7_^-^* | *n_7_^+^* | *n_7_^-^* |  | |  |
| Requirement fulfillment coefficient: | | | *Кв_7_ = n_7_^+^/ n_7_* | *Кв_7_* | *-* |  | |  |
